# Gating System Optimization for EV31A Magnesium Alloy Engine Body Sand Casting

**DOI:** 10.3390/ma15134620

**Published:** 2022-06-30

**Authors:** Andrzej Kiełbus, Robert Jarosz

**Affiliations:** 1 Department of Materials Technologies, Silesian University of Technology, ul. Krasińskiego 8, 40-019 Katowice, Poland; 2 ZM “WSK Rzeszów” Sp. z o.o., ul. Hetmańska 120, 35-078 Rzeszów, Poland; jarosz.robert@zmwskrz.com

**Keywords:** magnesium alloy, EV31A, gating system, MAGMASoft, simulation, sand casting, foundry

## Abstract

The research presented in this paper aimed to change the existing gating system that would enable the engine body casting, from a new EV31A magnesium alloy, of the required quality. For this reason, the casting process simulations used the MAGMASoft software, followed by the experimental validation of the achieved results. The results achieved in the first stage of the cast computer simulation enabled the identification of potential problems and factors that reduce the casting quality. However, the proposed design modifications eliminated the inadequate delivery of liquid metal to the casting’s critical areas by adequately controlling the mold cavity filling and solidification process. The experiment validated the simulations of the computer casting defects at the various stages. The results enabled the new EV31A magnesium alloy to be implemented in industrial production.

## 1. Introduction

The development of structural components for the aerospace industry focuses on reducing weight while improving mechanical properties [1]. It increases the interest in the new generation of magnesium alloys in the design process of modern aircraft engines. These are alloys characterized by low density and good mechanical properties at elevated temperatures [2].

The previous experience shows the necessity of replacing the used magnesium casting alloys with new ones that offer better functional parameters. In the last decade, it has made most cast components for the aerospace industry from AZ91 magnesium alloy. Research of engine components made of this alloy, exploited for over two thousand hours, shows that they are susceptible to crack, mainly in areas where the service temperature was higher than 120 °C [3]. The mechanical properties of the AZ91 alloy significantly decrease due to the Mg_17_Al_12_ phase precipitates at α-Mg solid solution grain boundaries [4,5].

Therefore, it was necessary to replace the AZ91 alloy with the Mg-Y-RE group alloy characterized by a service temperature of 200 ÷ 250 °C [6]. It involves fundamental changes in casting mold making and liquid alloy preparation [7]. However, these alloys are expensive (addition of neodymium and yttrium) [8,9]. The EV31A alloy, which contains gadolinium instead of yttrium, is a suitable, cheaper alternative to these alloys [10,11]. The unique properties of this alloy (fluidity, solidification rate) cause significant changes in the mold pouring and solidification process. Accordingly, technological testing and the design process verification of new casting technology are required [12].

The gating system design is a critical factor in the sand casting process, which not only directly influences the molten metal flow, heat transfer, and alloy solidification but allows better casting quality [12,13]. Improper gating system design causes an increased liquid metal flow rate. That results in more turbulence on the surface, which promotes oxide film entrapment inside the liquid metal [14,15]. These trapped oxide layers promote the formation of other casting defects, such as shrinkage porosity and cracks [16]. An ideal gating system reduces the turbulent flow of molten metal inside the mold cavity, holds gas to a minimum level, and limits impurities [17].

In contrast to other alloys, magnesium alloys are susceptible to oxidation and hydrogen absorption. In magnesium alloys, oxide formation is immediate. Therefore, the gating system design has a more significant role in minimizing the oxide ingress from the molten metal surface into the casting and also in preventing the liquid metal from turbulence, the flow free-falling when moving from one level to another, or the flow direction changing rapidly. Because of this, magnesium castings are more susceptible to defects, such as porosity and oxide inclusions resulting from an incorrect gating system design [12].

Casting simulations using specialized software are an integral part of any process because they can significantly reduce production costs and product development time. The computer simulation used in the foundry industry is not a new idea. Many papers in the scientific literature aimed to get high-quality castings free from defects by understanding areas such as gating system design, filling and solidification sequence, and the influence of casting process parameters (thermophysical data, liquid metal temperature) [18]. However, each casting, not shaped differently but made of a dissimilar alloy, is an object that requires an alternative approach. Therefore, the gating system needs also to develop for each specific shape, size, wall thickness, and alloy. For this reason, increasingly advanced tools and computer programs are helpful in the gating system and casting mold design [19].

This paper presents the results of using MAGMASoft software (ver. 5.0, MAGMA Giessereitechnologie GmbH, Aachen, Germany) in the gating system optimization process of a helicopter engine body casting made of the EV31A magnesium alloy. Because of the new alloy’s different thermophysical properties, the previously used gating system design required changes. The liquid alloy flow simulation allowed the identification of the critical spots in the gating system and the mold cavity. Meanwhile, the casting solidification simulation allowed the selection of the optimum pouring temperature and the determination of the optimum casting supply. The article also contains a simulation results analysis regarding casting defect possibilities related to casting fill and mold pouring parameters. Because of the gating system modification, the casting has the required quality, while the porosity is less than 2%. X-ray results and microstructure evaluation in critical areas of the actual casting confirm this. The results enabled the new EV31A magnesium alloy to be implemented in industrial production.

## 2. Materials and Methods

### 2.1. Materials

The material used in this study was EV31A magnesium alloy. Table 1 shows the chemical composition of this alloy. It is an alloy used in the sand casting form mainly in the aerospace industry.

Table 2 shows the chemical composition of Mrs9Mg molding sand used to form the mold.

### 2.2. Simulation

**Figure 1:**
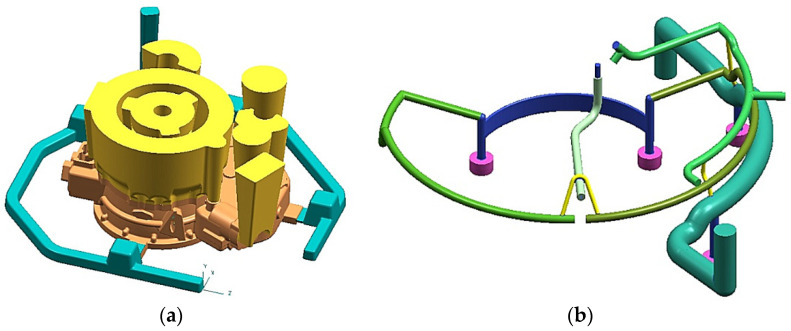
(**a**) 3D casting model with gating system; (**b**) 3D chromium-nickel tube collector model.

To create 3D solids of the investigated casting and casting mold, we used the Unigraphics NX2 program. Figure 1a shows an engine body model with 500 × 400 × 100 mm dimensions and 8 kg mass with the technological system used previously for AZ91 alloy. It is a layout with one shared distributor beam with a 35 × 30 mm cross-section area and four 45 × 10 supply gates. In addition, the gating system has typical open cylindrical risers feeding the casting’s primary thermal nodes. A cast iron chillers system placed in casting bottom areas, with a thickness of at least 10 mm, supports a directional crystallization process. Meanwhile, Figure 1b shows a chrome-nickel tube collector (fuel-oil lines) embedded in a casting.

The simulation used the SOLVER 5 version of the MAGMASoft software, whose mathematical model closely reflects the processes occurring during the mold cavity filling. Table 3 shows the material’s thermophysical properties used in the simulation.

### 2.3. Experimental

**Figure 2:**
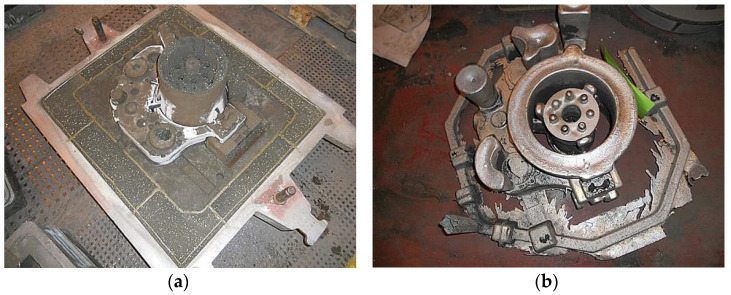
(**a**) Casting mold during assembly; (**b**) engine body sand casting with the gating system.

For the EV31A alloy, melts used an electric furnace with a maximum of 70 kg input. The protective gas mixture used was Ar-6 dm^3^/min, CO_2_-6 dm^3^/min, and SF_6_-0.16 dm^3^/min. Sand casting temperature was 760 ÷ 780 °C with stirring’s melt homogenization. Figure 2a shows the casting mold during assembly, while Figure 2b shows the engine body sand casting with the gating system.

### 2.4. XRD and Microstructural Examinations

**Figure 3:**
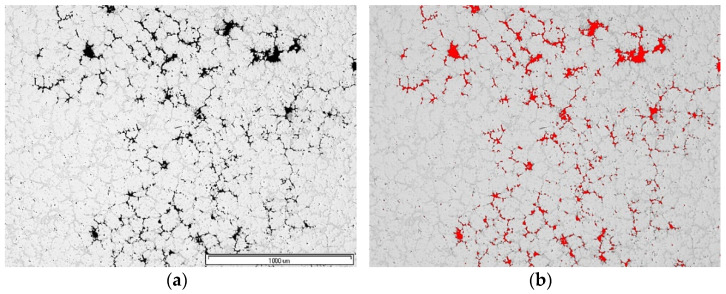
Porosity evaluation procedure: (**a**) gray input image; (**b**) detection, image for measurement.

The X-ray testing usedX5000 industrial CT X-ray inspection system (North Star Imaging, Rogers, MN, USA). From the highest porosity areas shown in the simulation, samples were cut for microstructure studies. Metallographic specimens used a typical procedure. The research used an Olympus GX71 optical microscope (Olympus, Tokyo, Japan) and a Hitachi S3400N scanning microscope (Tokyo, Japan). Meanwhile, the quantitative porosity analysis used the image analysis software AnalySIS Pro (AnalySIS Pro® ver. 5.0 Olympus, Tokyo, Japan) (Figure 3).

## 3. Results and Discussion

In the first research stage, the simulation included only the casting, the tube collector, the mold body, and the gating system used to cast AZ91 alloy. The chrome-nickel tube collector sunk in casting in MAGMASOFT is a chiller heated to about 80 °C before pouring. The first stage aimed to observe the mold filling process for liquid melt disturbances and turbulences with the secondary oxide inclusions formation. For the EV31A alloy, this is very important because of the zirconium’s high affinity for bonding with oxide inclusions. Zirconium concentration around oxide inclusions results in the formation of a structural defect and reduces the effect of the α-Mg solid solution grain refinement. However, the increased number of defects significantly affects the directional solidification process disturbance (casting, risers) and shrinkage defect formation.

**Figure 4:**
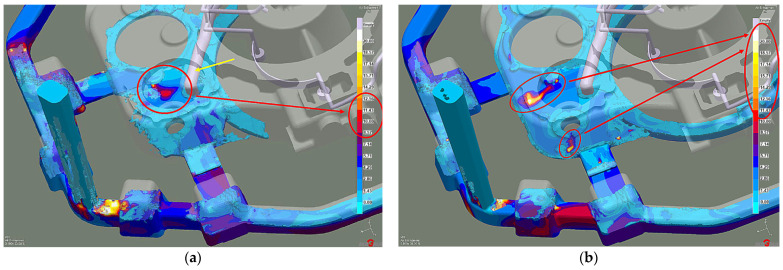
Stage 1 Liquid metal-air entrapment: (**a**) 20% of mold filling; (**b**) 35% of mold filling.

The significant factor affecting a liquid alloy’s intense oxidation in the mold cavity is the air content in the melt. The critical value is 20%, while the air volume preferred should not exceed 10%. When the mold cavity is 20% filled (Figure 4a), the liquid melt stream collision phenomenon is visible near the highest diameter collector tubes (yellow arrow). This effect causes a local air concentration to increase between 8.5% and 12.8%. However, when the mold cavity is 35% filled (Figure 4b), the liquid melt air content increases locally to over 20%. In addition, another higher air content area creates in the casting. The same is true of the other feeding gates (WD), with air content between 5% and 15%. However, as the liquid alloy mirror rises in the mold cavity, the air content decreases, or secondary oxides form.

**Figure 5:**
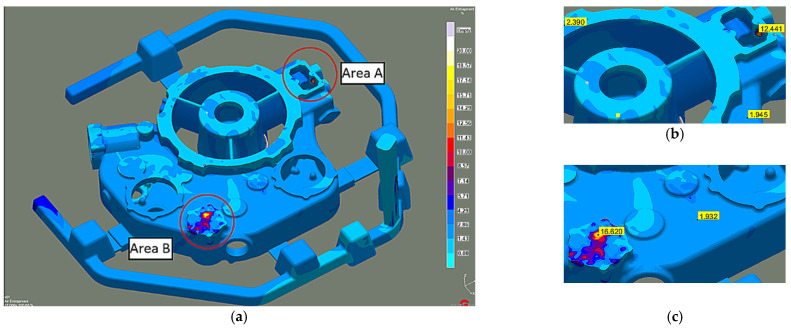
Stage 1 Liquid metal-air entrapment-100% of mold filling: (**a**) whole casting; (**b**) area A; (**c**) area B.

**Figure 6:**
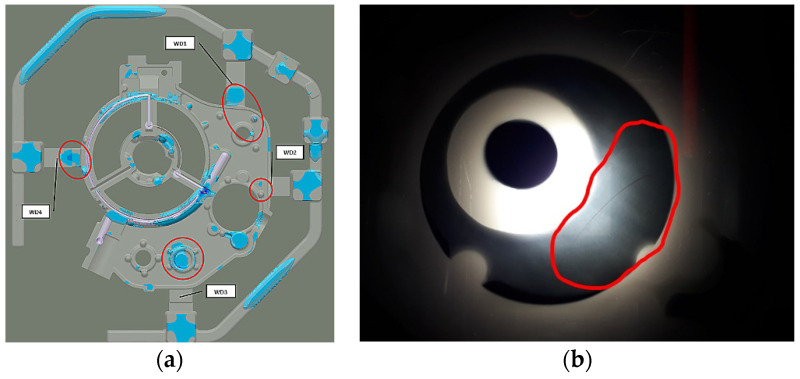
Stage 1 (**a**) Porosity in feeding gates areas; (**b**) Corresponding X-ray image.

On the upper casting walls, after 100% mold filling, the liquid melt air content is less than 3%. Two casting areas show increased amounts of air between 12% and 16% (Figure 5). However, these are the places where there will be risers feeding and thus exhausting the cold, oxidized, and air-rich liquid melt front. Meanwhile, the porosity (total porosity criterion), analyzed only in the liquid melt feed areas, was as high as 70% in the WD3 area (Figure 6a). In other zones, it is less than 2%.

**Figure 7:**
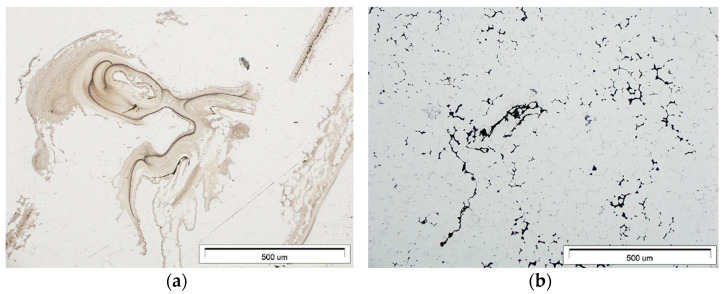
Stage 1 (**a**) Oxide impurities; (**b**) porosity in the areas marked in Figure 5a (LM).

The actual cast X-ray examinations confirmed the defects present in these areas (Figure 6b). These are mainly oxide impurities (Figure 7a) and significant porosity (Figure 7b), which reach ~30% in the areas marked in Figure 6a.

**Figure 8:**
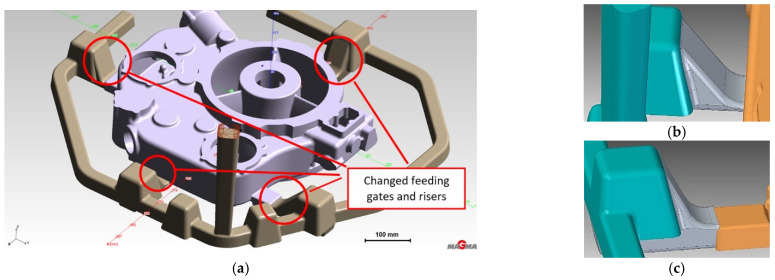
Stage 2 (**a**) Gating system with changed feeding gates and risers; (**b**) changed riser WD2; (**c**) changed riser WD4.

The second stage aimed to determine the increased feed cross-section effect on reducing turbulence and eliminating oxide inclusions in the liquid melt. In addition, the shape of the feeding gates at each gate changes, introducing a smooth transition with the entry radius into the gate’s top surface (Figure 8).

**Figure 9:**
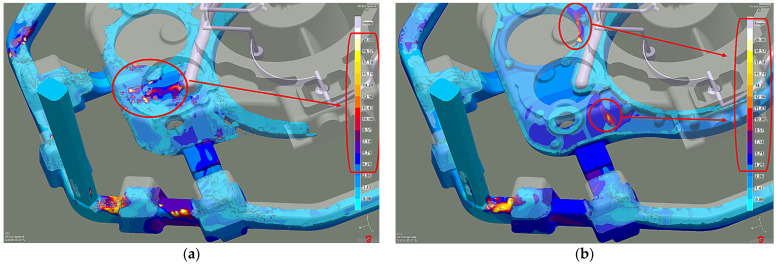
Stage 2 Liquid metal-air entrapment: (**a**) 20% of mold filling; (**b**) 35% of mold filling.

After these changes, at 20% mold filling, larger air bubble clusters occur in the liquid melt compared to the first stage (Figure 9a). The air volume in the centers of the clusters reaches values above 20%. However, during the successive filling of the mold cavity, the area number and the air volume in those areas decrease (Figure 9b).

**Figure 10:**
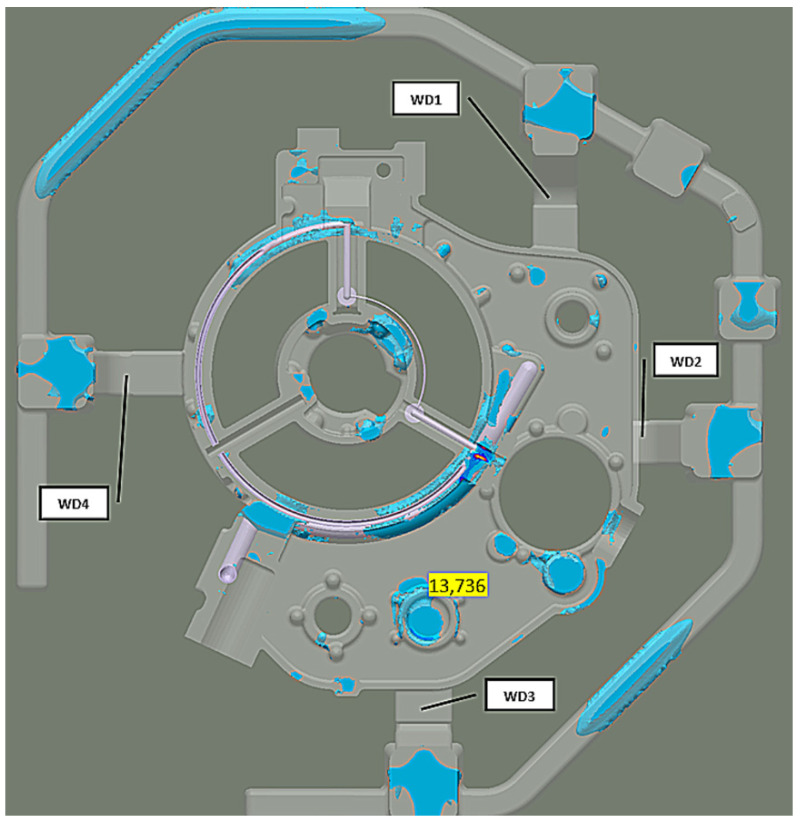
Stage 2 Porosity in the feeding gate areas.

The increased liquid melt flow rate through the individual feeding gates moves the air-liquid metal mixture into the casting regions where the hot spots (oil channels, flanges) occur. Less entrapped air reduces casting porosity. WD1 and WD4 feeding gates are defect-free. However, in the WD3 feeding gate, the porosity decreased from 70% to about 13% (Figure 10).

**Figure 11:**
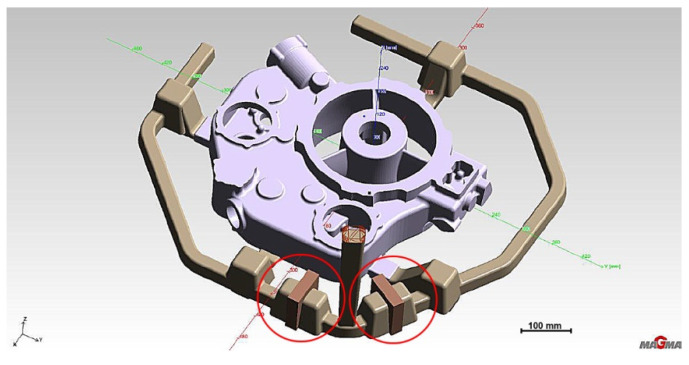
Stage 3 Changed filter system with ceramic filters.

**Figure 12:**
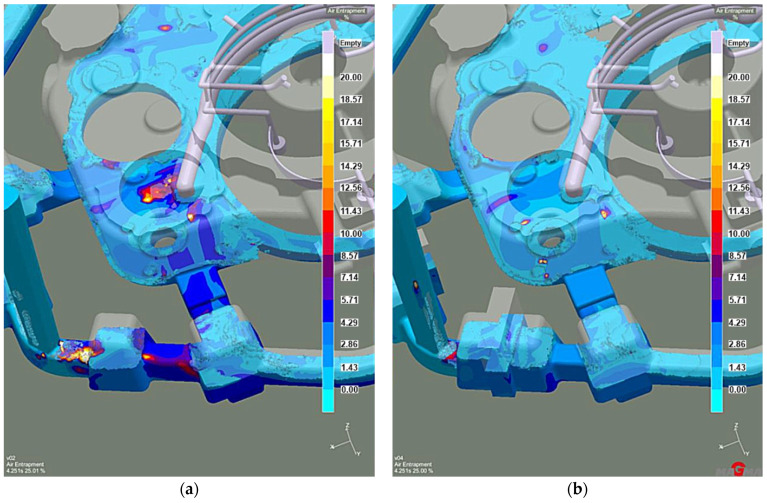
Stage 3 (**a**) Filter system without ceramic filters; (**b**) Filter system with ceramic filters.

In the next stage, larger slag tanks used ceramic filters with a 10 ppi density (Figure 11). The larger ceramic filter tank provides a smoothly liquid melt flow into the mold cavity, an air lower proportion in the liquid melt, and fewer air-trapped clusters (Figure 12).

**Figure 13:**
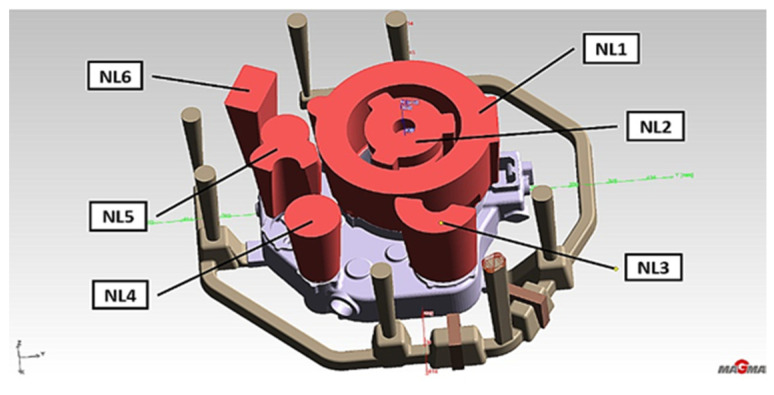
Stage 4 Feeding system through the gravity top risers.

The gravity top feeding system is used in the fourth stage of the simulation. Risers occur in the hot node’s main areas and exits of the oxidized liquid alloy front (Figure 13).

**Figure 14:**
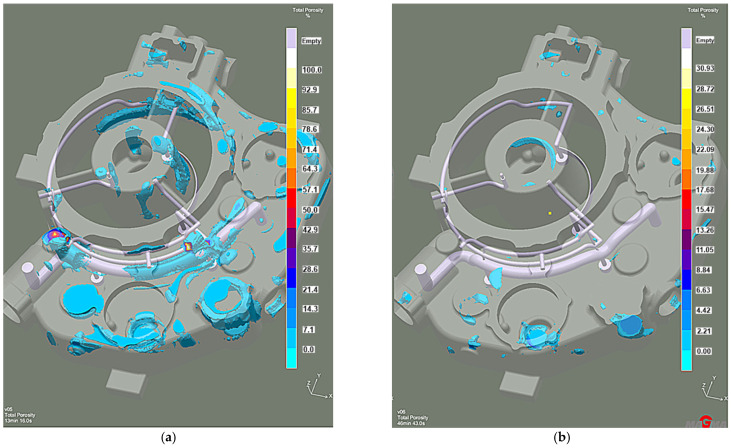
Stage 4 Total Porosity criterion: (**a**) the system without feeding elements; (**b**) the system with top feeding risers.

**Figure 15:**
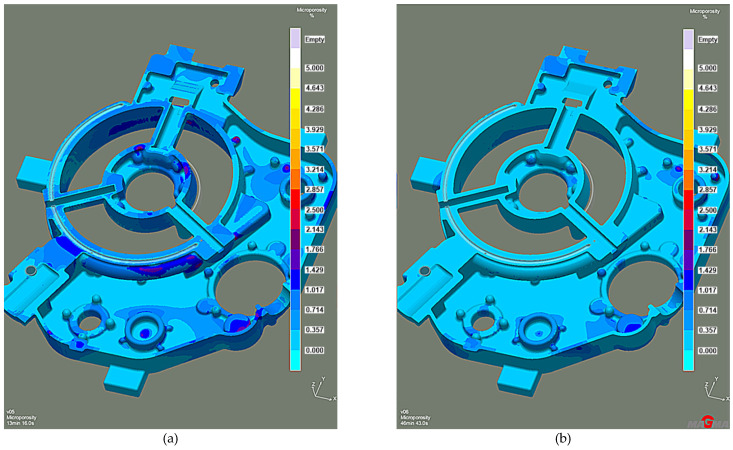
Stage 4 Microporosity criterion: (**a**) the system without feeding elements; (**b**) the system with top feeding risers.

Efficient feeding process analysis shows that top feeding risers eliminate casting shrinkage and reduce shrinkage porosity. Most of the resulting defects occur in the casting lower zones. However, these are zones cut off from the cast crystallization directional processes because of the casting geometry. This solution prevents the casting’s lower zones from feeding, as the feeding zones do not connect to the thermal node zones. The results of the comparison with the previous system without the feeding elements (Figure 14a) reveal that the introduced risers eliminate most shrinkage defects (Figure 14b). The same is true of the porosity. While the system without feeding elements has over 3% microporosity (Figure 15a), the system with top feeding risers has less than 1.5% microporosity (Figure 15b).

**Figure 16:**
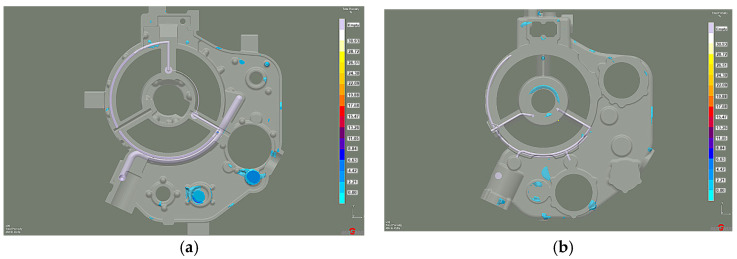
Stage 4 Porosity: (**a**) lower casting part; (**b**) top casting part.

**Figure 17:**
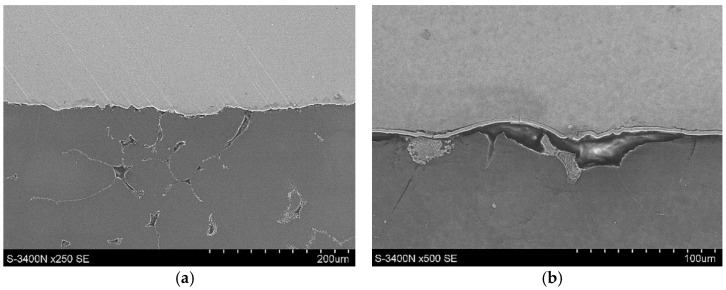
Stage 4 Casting-collector area: (**a**) porosity; (**b**) leakage (SEM).

The highest microporosity occurred in the chrome-nickel tube collector areas and at the feeding gates. Its presence contributed to the leak formation in the casting wall cross-section (Figure 16a). In addition, the lower casting parts show more porosity (Figure 16b). The microstructure examination of the cast near these areas (Figure 17) confirms this.

**Figure 18:**
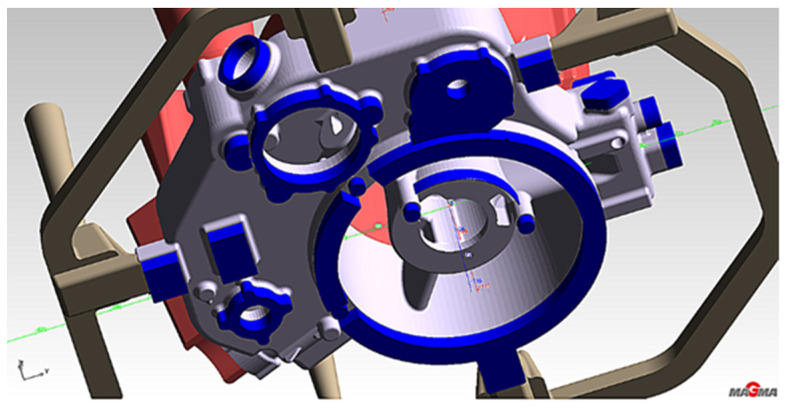
Stage 5 Cast iron chillers with increased thickness.

Alloy solidification process control, which creates one-directional crystallization favorable conditions, requires the addition of technological elements that increase the effect of the faster boundary phenomenon. Cast iron chillers are these elements, which, together with the risers, will create a stabilized crystallization process. Their correct size and shape allow a properly directed crystallization front. Therefore, in stage 5, the cast iron chiller’s thickness increased from 10 mm to 15 mm (Figure 18).

**Figure 19:**
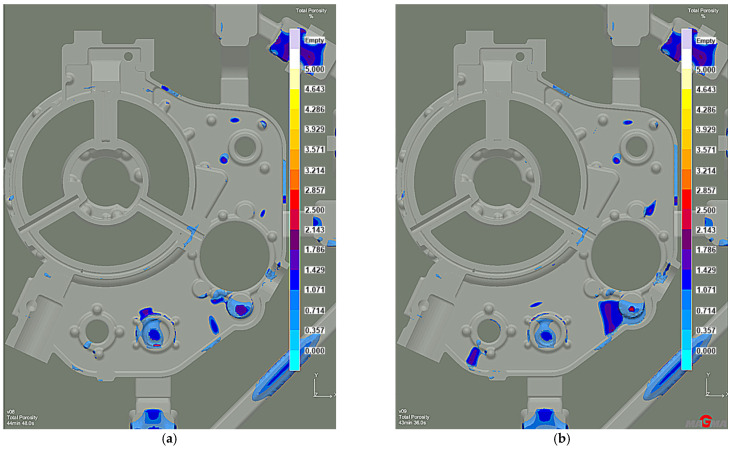
Stage 5 Total Porosity criterion: (**a**) 10 mm chiller thickness; (**b**) 15 mm chiller thickness.

**Figure 20:**
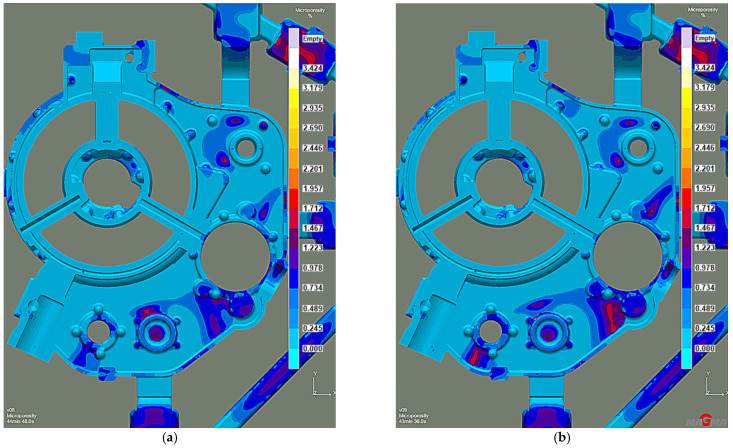
Stage 5 Microporosity criterion: (**a**) 10 mm chiller thickness; (**b**) 15 mm chiller thickness.

The distribution of shrinkage defects (Total Porosity criterion) shows less intensity and location with the lower chiller thickness version (Figure 19), while the porosity moved from the machined surfaces into the casting walls. However, it is insignificant and remains below 2% (Figure 20). Therefore, increasing the chiller’s thickness can be the preventive protection against mold overheating caused by the permissible pouring temperature exceeding.

**Figure 21:**
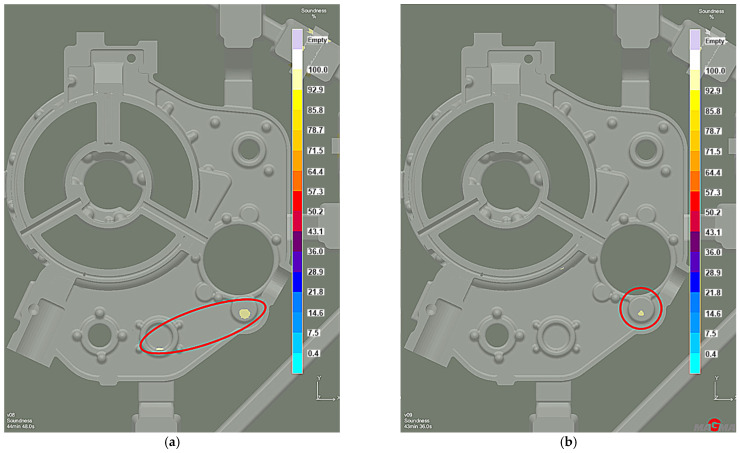
Stage 5 Soundness criterion: (**a**) 10 mm chiller thickness; (**b**) 15 mm chiller thickness.

**Figure 22:**
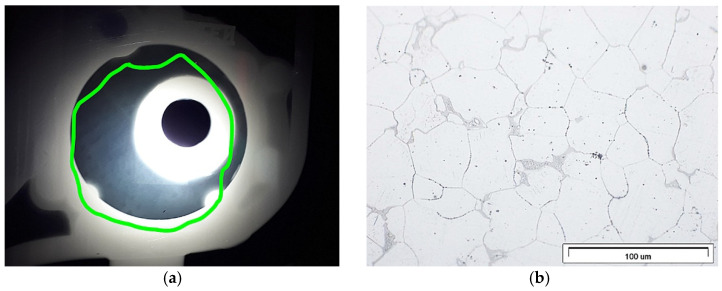
Stage 5 Casting critical areas: (**a**) XRD results; (**b**) low porosity (LM).

In addition, the shrinkage porosity significantly decreased (Figure 21), which confirmed the XRD results (Figure 22a) and microstructure research (Figure 22b).

## 4. Summary

The research presented in this paper aimed to change the existing gating system that would enable the engine body casting, from a new EV31A magnesium alloy, of the required quality. For this reason, the casting process simulations used the MAGMASoft software, followed by the experimental validation of the achieved results. The engine body casting process computer simulation results conducted in the first stage enabled the identification of potential problems and factors that reduce EV31A magnesium alloy casting’s quality. The proposed design modifications eliminated the inadequate liquid metal delivery to critical areas of the casting by adequately controlling the mold cavity filling and solidification process. Design corrections included:

- 2nd stage: increase the feed cross-section and feeding gates shape on each gate, introducing a smooth transition with the entry radius into the top gate top surface, which reduces turbulence and eliminates oxide inclusions
- 3rd stage: introducing the ceramic filters, which should protect the casting from impurities
- 4th stage: using a gravity top feeding system, which compensates for casting shrinkage and reduces porosity
- 5th stage: increase the chillers thickness to 15 mm, which reduces the shrinkage defects volume and microporosity to a maximum of 2%.

The experiment validated the computer simulations of the casting defects at various stages. The engine body, cast according to stage 5 guidelines, has almost no shrinkage porosity. However, the remaining micropores are magnesium alloy casting’s inseparable parts. Thus, the casting analyzed meets all requirements and is suitable for industrial production. Therefore, the system achieved in step 5 is the optimal technological system with a minimal tendency to microporosity based on both simulation and experimental results. The changed design of the gating system made it possible to implement the new EV31A magnesium alloy in industrial production.

## Figures and Tables

**Table 1:** Chemical composition of EV31A magnesium alloy (wt.%).

Nd	Gd	Zr	Zn	Mn	Fe	Ag	Mg
2.7	1.2	0.49	0.4	0.001	0.003	0.01	Balance

**Table 2:** Chemical composition of Mrs9 molding sand (kg).

Quartz Sand	ASKURAN 040 Resin	HARTER GS20 Hardener	Granulated Sulfur	Boric Acid	Potassium Fluoroborate
82	6.5	3.0	4.0	2.0	2.5

**Table 3:** The thermophysical properties of the material used in the simulation.

Material	Initial Temperature (°C)	Density (kg/m^3^)	Thermal Conductivity (W/mK)	Specific Heat (J/kgK)
Chill	20	7850	29	700
Molding sand	20	1500	0.9	741
EV31A alloy	T_solid_ = 523 T_liquid_ = 625	1835	118.3	1049

## Data Availability

Not applicable.

## References

[1] Luo A.A. (2013). Applications: Aerospace, Automotive and Other Structural Applications of Magnesium. Fundamentals of Magnesium Alloy Metallurgy: A Volume in Woodhead Publishing Series in Metals and Surface Engineering.

[2] Mordike B.L., Ebert T. (2001). Magnesium Properties—Applications—Potential. Mater. Sci. Eng. A.

[3] Luo A.A. (2004). Recent Magnesium Alloy Development for Elevated Temperature Applications. Int. Mater. Rev..

[4] Moreno I.P., Nandy T.K., Jones J.W., Allison J.E., Pollock T.M. (2003). Microstructural Stability and Creep of Rare-Earth Containing Magnesium Alloys. Scr. Mater..

[5] Von Buch F., Mordike B.L. (2004). High-Temperature Properties of Magnesium Alloys. Magnesium—Alloys and Technology.

[6] Mordike B.L. (2002). Creep-Resistant Magnesium Alloys. Mater. Sci. Eng. A.

[7] Luo A.A. (2013). Magnesium Casting Technology for Structural Applications. J. Magnes. Alloy..

[8] Aghion E., Bronfín B., von Buch F., Schumann S., Friedrich H. (2003). Newly Developed Magnesium Alloys for Powertrain Applications. JOM.

[9] Bronfin B., Moscovitch N. (2006). New Magnesium Alloys for Transmission Parts. Met. Sci. Heat Treat..

[10] Lyon P., Syed I., Heaney S. (2007). Elektron 21—An Aerospace Magnesium Alloy for Sand Cast and Investment Cast Applications. Adv. Eng. Mater..

[11] Kiełbus A. (2011). Microstructure and Properties of Elektron 21 Magnesium Alloy. Magnesium Alloys—Design, Processing and Properties.

[12] Sun Z., Hu H., Chen X. (2008). Numerical Optimization of Gating System Parameters for a Magnesium Alloy Casting with Multiple Performance Characteristics. J. Mater. Processing Technol..

[13] Peng Y.H., Li D.Y., Wang Y.C., Yin J.L., Zeng X.Q. (2005). Numerical Study on the Low Pressure Die Casting of AZ91D Wheel Hub. Mater. Sci. Forum.

[14] Katzarov I.H. (2003). Finite Element Modeling of the Porosity Formation in Castings. Int. J. Heat Mass Transf..

[15] Dai X., Yang X., Campbell J., Wood J. (2003). Effects of Runner System Design on the Mechanical Strength of Al–7Si–Mg Alloy Castings. Mater. Sci. Eng. A.

[16] Cáceres C.H., Selling B.I. (1996). Casting Defects and the Tensile Properties of an AlSiMg Alloy. Mater. Sci. Eng. A.

[17] Ahmad R., Hashim M. (2011). Effect of Vortex Runner Gating System on the Mechanical Strength of Al-12Si Alloy Castings. Arch. Metall. Mater..

[18] Khan M.A.A., Sheikh A.K., Asad M. (2020). Mold Design and Casting of an Impeller Using MAGMASoft. Int. J. Mech. Eng. Robot. Res..

[19] Jezierski J., Dojka R., Janerka K. (2018). Optimizing the Gating System for Steel Castings. Metals.

